# Ethics, One Health approaches, and SDGs: conference lessons for an emerging field

**DOI:** 10.3389/fpubh.2024.1448409

**Published:** 2024-12-11

**Authors:** Henrik Lerner, Rebecca E. Nordquist, Zohar Lederman, Jared Keyel, Patricia Mooney Nickel, Charlotte Berg

**Affiliations:** ^1^Department of Health Care Sciences, Marie Cederschiöld University, Stockholm, Sweden; ^2^Unit Animals in Science and Society, Department of Population Health Science, Faculty of Veterinary Medicine, Utrecht University, Utrecht, Netherlands; ^3^Department of Emergency Medicine, Li Ka Shing Medical Faculty, Hong Kong University, Hong Kong, China; ^4^Centre for Medical Ethics and Law, Hong Kong University, Hong Kong, China; ^5^International Center of Health, Law, and Ethics, University of Haifa, Haifa, Israel; ^6^Department of Sociology and Anthropology, Rowan University, Glassboro, NJ, United States; ^7^Center for Public Health Practice, Colorado School of Public Health, Aurora, CO, United States; ^8^School of Social and Cultural Studies, Victoria University of Wellington, Wellington, New Zealand; ^9^Department of Applied Animal Science and Welfare, Swedish University of Agricultural Sciences, Skara, Sweden

**Keywords:** One Health approaches, risk, resilience, wicked problems, dialogue ethics, interdisciplinarity

## Abstract

One Health ethics is an emerging field that has gained traction since its origin in approximately 2015. This article builds upon the insights shared during a panel discussion on *One Health, Sustainable Development Goals (SDGs), and ethical conflicts* at the 28th Annual International Sustainable Development Research Society Conference. The conference, themed *Sustainable Development and Courage: Culture, Art, and Human Rights,* aimed to advance and expand recent knowledge in the field. Key themes discussed during the conference panel included interdisciplinarity and multidisciplinary, risk, resilience, wicked problems with no readily available solutions, and *praxis*. A conclusion is that ethics should become more prominent within One Health discussions. Four aspects emerged from this discussion: (1) Ethics is needed to solve wicked problems within One Health approaches. (2) Aspects of multi-, inter-, and transdisciplinarity need to be considered together with their implications for ethics. (3) Two crucial concepts, risk and resilience, need to be addressed. (4) Ethical decision models are called for and need to be developed.

## 1 Introduction

The One Health approaches (1) have been a valuable area of bioethics research due to their strong multispecies perspective and inter- or transdisciplinary nature, both scientifically and societally. Analyzing these approaches requires a wide scope, as they relate to medical, animal, and environmental ethics. The field of One Health ethics is still in its infancy.

To advance and deepen the integration of ethics within One Health approaches, the 28th Annual ISDRS^1^ Conference, *Sustainable Development and Courage: Culture, Art and Human Rights,* featured a dedicated track on *One Health, SDGs, and ethical conflicts*, where “SDGs” is the abbreviation of the United Nations Sustainable Development Goals.^2^ The track brought together presenting scholars from the Netherlands, Israel, Sweden, and the USA to share thoughts and scrutinize the ethical aspects of One Health. The conference participants have composed this article to present aspects of the ethics discussion that took place at the conference and to explore future directions for ethics in One Health.

## 2 History and initial themes of ethics

The *One Health, SDGs, and ethical conflicts* track began with an overview of the history of ethics within One Health approaches. Since approximately 2015, One Health ethics has focused on three areas: (1) developing ethical frameworks (2–4), (2) analysis of values (1, 2, 5, 6), and (3) addressing ethical issues (7, 8). The 2022 conference in Stockholm contributed to this field by analyzing concepts and principles, such as risk and security, and ethical methods, such as dialogue and decision models. Given the broader conference theme, this panel focused on integrating One Health approaches and the SDGs, specifically focusing on at least 11 of the 17 SDGs. Followed by the talks from the panelists, there was a workshop discussion on ethics related to One Health approaches. The following sections summarize panelist contributions and propose directions for future research in One Health ethics.

## 3 Risk, resilience, and the importance of interdisciplinary approaches

Rebecca Nordquist focused on the concepts of resilience and risk. Her presentation highlighted that One Health inherently necessitates multidisciplinary or interdisciplinary collaborative teams. To recognize the overlaps in health between humans and non-human animals, insights are needed from fields such as veterinary health, human health, epidemiology, human and animal behavior, toxicology, and ethics.

In a multidisciplinary approach involving researchers from various faculties at Utrecht University in the Netherlands, there was an identification of a potential transition to a (more) circular agricultural system as a wicked problem with issues related to the One Health domain. Food and agricultural system transitions align closely with classical definitions of a “Wicked Problem”: the problem itself is difficult to define, there are no true/false answers to the problem, and there is no clear solution to the problem or even a clear possible indicator that the problem has been solved (9, 10). Food and agricultural system transitions also largely fit definitions of “super wicked problems” proposed, most poignantly, there is no central authority to tackle the problem (10), which immensely complicates decision-making processes. With food and agriculture systems, changing one part of the equation will have far-reaching effects in fields outside of the individual researchers’ familiarity. This implies that individual researchers may overlook or underestimate risks outside their specific area of expertise.

Food and agriculture systems are strongly linked to One Health, as the welfare of humans who rely on safe and reliable food is linked to the welfare of animals kept as livestock and animals affected by alterations of ecology due to food production. Farmers who keep livestock and companies that are economically relevant in food production are linked to animal health and disease through zoonoses and the economic impacts of animal disease. Farms and farming areas are linked to the human health of residents in proximity to farms through, among others, air quality (11).

Interdisciplinary research is necessary in a context as complex as One Health, but by no means easy. True interdisciplinarity requires conscious efforts from all parties involved to avoid misunderstanding and to reach beyond simply piecing together information from several fields. A process is required to designate relations established between elements that were not previously related, a point termed integration (12). In examining the transition to circular agriculture from a multidisciplinary perspective, we first encountered issues surrounding definitions when working toward interdisciplinarity. One of our focus areas is the risk that such a transition may entail: risk to food security, animal welfare, public health, and governance, to name a few. Although all partners discussed the “risk” of a transition, it became clear that we were all using the term differently. Risk and risk assessment can be used in different contexts across fields. A few examples from various relevant disciplines that have been found are:

• Chemical risk/toxicology:
 o “Chemical risk assessment is the process of identifying and characterizing the potential adverse consequences of chemical exposures. This process is typically divided into the four components of exposure assessment, hazard identification, dose–response assessment, and risk characterization” (13).
• Environmental sciences and natural disaster preparedness:
 o “We define risk as the product of hazard, exposure, and vulnerability, where a hazard is a phenomenon that causes impact to exposure, assets, and people in harm’s way, depending on their vulnerability” (14).
• Risk assessment for animal welfare guidance:
 o “Risk: A function of the probability of negative welfare consequences and the magnitude of those consequences, following exposure to a particular factor or exposure scenario, in a given population” (15).
 o “Risk assessment has three elements: exposure assessment, consequence characterization, and risk characterization. Exposure assessment should provide a qualitative or quantitative evaluation of the strength, duration, frequency, and patterns of exposure for the factors relevant to the exposure scenario(s) developed during the problem formulation” (15).

Within our interdisciplinary team, similar issues in definitions of terms arose surrounding the term “resilience.” Transitioning food systems to more sustainable systems entails changing the risk levels currently associated with food systems. This could negatively influence humans and animals regarding food safety, food security, environmental issues, governance, and more. One way to mitigate the risks involved in changing a complex system like our food system is to work toward a resilient system. Broadly, this system can deal with change and still perform primary functions, i.e., providing safe and healthy food. Both recognition of potential risks and building toward resilience are essential to foster a shift to sustainable food systems.

All involved in the interdisciplinary project agreed that to support transition, a resilient food system is needed. However, each discipline defines resilience differently. The following are a few examples from the disciplines involved:

• Resilience management from a systems perspective: (7 principles of resilience management)
 o Maintain diversity and redundancy
 o Manage connectivity
 o Manage slow variables and feedback
 o Foster an understanding of social-ecological systems as complex adaptive systems
 o Encourage learning and experimentation
 o Broaden participation
 o Promote polycentric governance systems (16).
• Resilience from a psychology perspective:
 o “Most definitions are based around two core concepts: adversity and positive adaptation” (17).
 o An article at a conference with the International Society of Traumatic Stress Specialists (18) concludes that defining is difficult but that most panelists included “most of the proposed definitions included a concept of healthy, adaptive, or integrated positive functioning over the passage of time in the aftermath of adversity.”
 o APA online dictionary: “the process and outcome of successfully adapting to difficult or challenging life experiences, especially through mental, emotional, and behavioral flexibility and adjustment to external and internal demands.”^3^
• Biological resilience definitions tend to include stress:
 o “The term “resilience” refers to the ability to adapt successfully to stress, trauma, and adversity, enabling individuals to avoid stress-induced mental disorders such as depression, posttraumatic stress disorder (PTSD), and anxiety” (19).
 o “Resilience is the ability to adapt successfully in the face of stress and adversity” (20).

To progress from multidisciplinary work to an interdisciplinary project toward the point of integration, as we aspire to do, much care is needed in the use and definitions of terms. It may not be possible, or even desirable, to reach a single, all-encompassing definition as a first step; this can take so much time and effort that it hinders progress toward real-world solutions (21). However, even if a single definition that covers all fields is not reached, at minimum, all involved in the project need to be aware of the differences in definitions.

Risk is a central concept in our project; thus, some consensus was necessary to facilitate discourse. We settled on a broadly taken definition: “hazard x exposure is risk”; in (working) definition, risk implies the presence of something that could potentially cause harm (a hazard), but the amount of exposure to the hazard is an essential part of the equation. These risks can be health-related risks, environmental risks, risks to food safety, or other types of risks. These will influence both humans and animals from a One Health perspective. Within the ethical matrix of One Health, the acceptable risk will need to be considered.

## 4 Solidarity and One Health in the age of COVID-19

Zohar Lederman talked about solidarity and One Health in the age of COVID-19. The COVID-19 pandemic highlighted instances of human solidarity, where individuals bore burdens to support others with shared circumstances (22). Unfortunately, it has also revealed numerous instances of failed solidarity nationally (23) and internationally (24).

However, solidarity among fellow humans is not the only kind of solidarity to be considered. More-than-human solidarity means human empathy and care toward animals, including pets, farm animals, and wild animals (25–27).

The One Health literature discusses shared risks and benefits to express the idea that animals and humans may be susceptible to similar environmental risks, such as toxicants and zoonotic diseases. Consequently, public health and biomedical interventions such as vaccinations originally aimed at humans may benefit animals and vice versa. Normatively, then, policies and interventions that may have the added value of benefiting multiple species in a One Health spirit should be prioritized over those that stand to benefit only one species, namely humans (8, 28–30).

Using solidarity as a framework for identification-motivated assistance (31), these concepts of shared risks and benefits establish a basis for humans and animals to relate to one another: we all share risks and benefits to some degree and, therefore, should act for one another to reduce the former and increase the latter. Resilience, understood as the capacity to adapt and survive in lieu of an external stressor, may also be used here as a basis for identification. Both humans and animals share the need to optimize their resilience in the face of an increasingly unsafe environment (due to climate change and so on) and, therefore, should carry costs to optimize each other’s resilience.

Only moral agents can “identify” with anyone in a sense meant here, and these lead to two options: One option is that the term may be understood metaphorically in the case of non-moral agents. A second option is that only agents will be included in and of themselves in our normative deliberations (32). Whatever the case might be, these notions suggest a normative commitment to prioritize the welfare of other species, even at a cost to one’s own. Such a commitment, while theoretically valid to all agents—metaphorically or otherwise—obviously can only practically apply to moral agents who have the capacity to act upon such commitments. Dolphins may be limited moral agents as they are highly intelligent animals, even capable of reflexivity (33), but we cannot hold them accountable for voluntarily disrespecting codes of ethics.

Inter-human solidarity implicitly grounds many, if not all, the SDGs. Using a One Health language allows us to link the SDGs to more-than-human solidarity. This may mean much greater responsibility toward animals, as they constitute a vulnerable population. This theoretical reframing might have corresponding duties, for instance, by committing to provide clean water to both human and animal populations or by assuring sustainable environments in which both humans and animals dwell (34).

## 5 Procedure to dialogue

Jared Keyel and Patricia M. Nickel suggested that, as an ideal, One Health is already a potential ethical framework through which to achieve the SDGs. They analyzed the limitations of recent One Health discourse through its narrow epistemological lens (35). Keyel and Nickel established *praxis* as their methodological approach, arguing that it could broaden this lens. They also explored the tensions of sectoral thinking in One Health and the SDGs, and especially how the operationalization of these ideals may occlude their emancipatory potential. Keyel and Nickel conclude that understanding One Health as *praxis* and the SDGs as a potential effort toward One Health would potentiate a “thicker” dialogue that resists the tendency to depoliticize the ethical challenges encountered by both frameworks.

As multi-, inter-, and/or transdisciplinary endeavors, both One Health and the SDGs make knowledge claims according to sometimes conflicting epistemological perspectives. David Waltner-Toews (36) has characterized this situation as a “wicked problem”: “situations that can be defined from a variety of apparently incompatible perspectives” (p. 3). Faced with this situation, which “cannot, by definition, be resolved by gathering more data” (p. 7), Waltner-Toews (36) proposes that One Health ought to focus on the creation of public spaces for “creative, constructive, and high-quality conflict.”

For Keyel and Nickel, *praxis* builds on Waltner-Toews’ suggestion of solving wicked problems through an expanded understanding of the impact of social and epistemological relations on dialogue. Although Waltner-Toews is committed to dialogue, research on public participation in the policy process has demonstrated that these spaces are potentially characterized by depoliticized dialogue (37–43). Deliberative spaces risk devolving into “useful legitimating devices for an already-decided policy” (44, p. 9). Like other public spaces, the interpretation of One Health takes place in the context of “[differentiated] social groups with unequal status, power, and access to resources, and traversed by pervasive axes of inequality… The means of interpretation and communication in these societies are also stratified” (45) (p. 296). As demonstrated below, the unequal distribution of discursive resources is compounded by operational discourses, which potentially close discursive space and thereby depoliticize dialogue about public problems.

Operational discourses, such as definitions of One Health advanced by global governance organizations, often function according to socially constructed sectoral boundaries between “political” and “administrative” [see (46)] or “social” and “economic.” The transformative potential of the One Health ethos is its departure from this type of sectoral thinking. Defining the boundaries of dialogue is powerful because, as Freire noted, dialogue can transform reality (47) (p. 87). At the core of the concept of *praxis* is the idea that thought and action are intertwined and that ethical ideals cannot be divorced from practice. One of Freire’s key insights was to recognize that through dialogue we transform assumptions about what is possible.

In thinking about One Health as an ethos based on *praxis,* Keyel and Nickel hope to integrate these critical perspectives on unequal discursive resources and sectoral thinking with Freire’s observation that “[d]ialogue characterizes an epistemological relationship” (47) (p. 379). One Health conceived of in this way values dialogue that can address epistemological assumptions so that those who live with the consequences of oppressive knowledge practices can envision what Freire called the “untested feasibility” of change (47) (p. 102). Approaching One Health as *praxis* makes no distinction between theory and action (47) (p. 128). Understood through the lens of *praxis*, theorizing in dialogue with others is already a practice of collaboration. Shared understanding matters because we act according to how we understand the world. This is not to say that One Health practitioners do not need agendas, plans, budgets, management coordination, and other tools for “getting things done.” However, movement between theory and action need not be made up of discrete linear steps.

Keyel and Nickel focus on the practice of One Health at the level of global governance, as reflected in the One Health High-Level Expert Panel (OHHLEP). They identify two key epistemological statements that, in their framing, resist dialogue and thus inhibit the transformation of our assumptions about what is possible for One Health. It is significant that, in their framing, the texts seem to avoid the appearance of “incompatible perspectives” observed by Waltner-Toews (36). Keyel and Nickel argued that a “thin” discussion of needs often implicitly assumes that the politics of needs concerns only whether or not various predefined needs will or will not be provided for (45) (p. 293) but avoids the difficult question of *how* these needs will be provided for. However, a “thicker” engagement of the One Health principles would require that we make “contextual and controversial” decisions (45) (p. 293). For example: What does “equity between sectors and disciplines” require? This question is fundamental when considering multi-, inter-, or transdisciplinary approaches. Would “inclusion and engagement of communities and marginalized voices” to achieve “sociopolitical and multicultural parity” extend beyond voice to decision-making power?

Following this analysis, Keyel and Nickel proposed a preliminary Toolbox Dialogue (48) to explore the epistemological assumptions of One Health that would be contestable through One Health *praxis*. Looney et al. (48) argue that semi-structured philosophical dialogue enhances scientific cross-disciplinary communication. Their Scientific Research Toolbox Instrument uses survey prompts to uncover assumptions that frame research practice. Keyel and Nickel’s preliminary Toolbox Dialogue for One Health and the Sustainable Development Goals adapts Looney et al. (48) Scientific Research Toolbox Instrument.

Keyel and Nickel concluded by suggesting that framing questions facing One Health as “transformative problems” characterized by uncertainties and complexities that prompt ongoing dialogue about the role of science in society, our relationship with animals and the environment, and a deeper exploration of our assumptions about what is possible in the future. They argue that a *praxis* of One Health creates space to contest depoliticized versions of both One Health and the SDGs. Building on Waltner-Toews’ (36) call for “creative, constructive, high-quality conflict,” Keyel and Nickel’s preliminary Toolbox Dialogue for One Health endeavors to create space to challenge the epistemological and ontological assumptions of One Health and the SDGs in dialogue with others.

## 6 Ethical decision models

Henrik Lerner focused on pluralistic ethical decision models to solve ethical dilemmas that arise from wicked problems within One Health approaches. This is due to the various versions of ethical perspectives that may be present, such as anthropocentrism, biocentrism, or ecocentrism, within One Health approaches. Furthermore, one must consider several kinds of species as well as individual, population, and ecosystem levels of analysis. Multi-, inter-, or transdisciplinary aspects are also important to include and consider.

Two dilemmas were used to analyze three ethical methods of dilemma-solving. The first dilemma was parasites in ecosystems, which considers the conflict between biodiversity and disease eradication. Some (49) argue that healthy ecosystems include parasites, while others might recommend eradicating the parasite. The second dilemma considered animal-assisted interventions, where the dilemma is to solve ethical conflicts that might arise from the human-animal interaction, the view of the animal (instrumentalist vs. co-therapist), and that not only physical health is present, but mental and social human health as well as animal welfare matters (50).

The three ethical methods of solving dilemmas were the ethical matrix (51), wide reflective equilibrium (52), and the map method (53).

Mepham’s ethical matrix is a deliberation of three ethical principles (of wellbeing, autonomy, and justice) with the inclusion of several stakeholders (here, humans, animals, and ecosystems). The decision is based on scientific facts and case-by-case weighing of ethical conflicts (51).

Wide reflective equilibrium is based on reflected, well-grounded principles. Principles chosen by competing ones must be made by reasonable people, and the principles chosen should be such that reasonable people can sustain their commitment to them. Principles can be chosen from morals, general social theory, or religious views (52).

In the map method, one needs to analyze the case from all the different perspectives of environmental ethics to make a well-founded decision. Suggested perspectives by Kronlid (53) included intragenerational anthropocentrism, intergenerational anthropocentrism, sentientism, animal rights, biocentrism, and ecocentrism. For each of them, one needs to write down how one would reason in the case analyzed. These rather thick descriptions provide an insight into the complexity of the matter. When all perspectives are considered, one can then make a final decision that is at least well-informed.

Analyzing these three methods with the role of parasites in ecosystems yielded the following insights. In the ethical matrix, one has to consider at least affected species, non-affected vector species, and parasites with their wellbeing, autonomy, and justice. However, the three principles will not cover enough ethical matters to work within the One Health approaches. For the wide reflective equilibrium, there is a risk that ecological issues might be less considered if one looks at the emphasis on humans, animals, and the environment in One Health approaches. In the map method, all perspectives with their thick descriptions must be considered, covering, for example, risks and aspects of resilience.

For animal-assisted interventions, the ethical matrix identified key stakeholders to at least one client, one caregiver and one participating animal at a minimum. The wide reflective equilibrium seems relatively easy to reach when only humans and animals are involved. In the map method, thick descriptions for anthropocentrism, sentientism, and animal rights. Biocentrism and ecocentrism are less influential.

Insights from this attempt highlighted the strengths and limitations of the three perspectives. The ethical matrix, while useful, may lack the depth needed to thoroughly analyze dilemmas in One Health. The effectiveness of the wide reflective equilibrium heavily relies on the criteria for what constitutes “reasonable persons” in terms of discourse and values. The map method requires a holistic approach, making it more aligned with the multifaceted nature of One Health. However, if applied too simplistically, it risks creating the impression of addressing the entire issue without fully doing so.

## 7 Discussion (from the starting point to analysis of emerging themes)

Four themes emerged from the conference discussion. These were

1) Ethics is needed to solve wicked problems
2) Aspects of multi-, inter-, and transdisciplinarity need to be considered
3) Risk and resilience as crucial concepts in the ethics of One Health approaches
4) Ethical decision models are called for and need to be developed

We will further discuss these one by one.

### 7.1 Ethics is needed to solve wicked problems

There have been several calls for ethics within One Health approaches, and this article is one in a row to establish ethics within this scientific area. It is strange and surprising that ethics does not have a central place in One Health approaches as it has in some of its core sciences, such as human medicine, public health, nursing science, veterinary medicine, and animal welfare. From now on, ethics needs to be established within One Health.

### 7.2 Aspects of multi-, inter-, and transdisciplinarity need to be considered

One has to be aware that the strive within One Health approaches for multi-, inter-, and transdisciplinarity also might have implications for ethics. First, this simple claim is that ethics should be included within One Health approaches, as discussed in the previous section. In many research projects, ethics (due to its nature) must also be at the core alongside human medicine, veterinary medicine, and ecology. When local communities, including indigenous peoples’ populations, are involved in decision- and policymaking, the risk of making vulnerable populations even more vulnerable is minimized (54). Moreover, relevant cultural aspects would influence the ethical decision-making process.

Multi-, inter-, and transdisciplinarity might also imply that ethics should be viewed broadly, not just as ethics that apply to humans. Zohar Lederman has elsewhere argued that a definition of One Health has an inherent ethical imperative (55). Promoting health for humans, animals, and ecosystems must incorporate ethical considerations for all three groups, requiring a reconciliation of anthropocentric, zoocentric, biocentric, and ecocentric perspectives.

### 7.3 Risk and resilience as crucial concepts in the ethics of One Health approaches

Humans and non-human animals share multiple environments and face similar health risks. In the Anthropocene and amid climate change, these risks include extreme weather events such as heat waves, droughts, floods, biodiversity loss, food insecurity, and antimicrobial resistance. To survive these challenges, both humans and animals must remain resilient. The shared vulnerability to these risks and the mutual need for resilience engenders a moral claim for solidarity that extends beyond the human sphere. While the implications of this solidarity will be elaborated elsewhere, it is essential to further explore the concepts of risk and resilience here.

Both the biomedical and bioethical literature mentioned the idea of shared risks as a foundation for a biomedical claim and a normative claim. The biomedical claim is that more attention should be paid to research and public health policies devoted to environmental risks shared by humans and animals because it promises to be the most cost-effective strategy. The normative claim is that more attention should be devoted to the implications of the acknowledgement of shared risks because (1) some of these risks are man-made, and therefore humans have an obligation to address them in animals, and (2) the claim made above, that this idea of shared risks can potentially serve as a basis for human obligations toward animals as an offshoot of solidarity.

Despite its relevance to One Health, the philosophical exploration of risk remains limited. Risk involves the possibility of, but uncertainty about, an unwanted event. It is sometimes used to denote an event that may or may not happen and sometimes a cause for such an event. It may simply refer to the probability of an unwanted event happening, or it can refer to the severity of such an event. Multiplying these two elements’ results in an expectancy value, which is perhaps the most common way the risk has been understood and used, e.g., in public health risk–benefit analyses. Insofar as it relates to an unwanted event, any risk assessment is value-laden. The extent of the event’s undesirability will determine the risk’s significance (56).

Various factors make risk inherently value-laden. In several instances, risk can relate to an unwanted event for which we have no good sense of probability. Moreover, we may not even know what the unknown event might be; thus, the risk becomes that of the unknown. Finally, whether an event is unwanted will sometimes depend on the particular context, which may differ from the one in which the risk is considered during decision-making. In all of these scenarios, values, or ethical judgment, replace the unknown or the uncertain (56).

One Health approaches that relies on shared risks of both humans and animals is normative by default. Even assessments of risk that seem strictly “number”-based have an evaluative component in them. Importantly, this evaluation is conducted by humans for themselves and animals, meaning that our perception of animals as bearers of moral value will pre-determine our perception of the nature and severity of risk. If indeed we perceive animals to be moral patients but without a voice, we may act as their representatives, granting them a voice. A risk is “shared,” then, in the sense that humans “together” with animals engage in a shared decision-making process, determining what constitutes risk and how to address it.

### 7.4 Ethical decision models are called for and need to be developed

For ethics to work in a research environment that is dominated by scientific research, applied ethics in the form of ethical decision models needs to be developed. To solve wicked problems in a multi-, inter-, and transdisciplinary context where one cannot assume that all involved have sufficient ethical competence, one has to provide easy-to-use ethical decision models. Due to the state of the art, these must not be too simplified. Wicked problems involve many different perspectives and ethical claims; hence, ethical decision models within One Health approaches must consider this.

The awareness of possible interspecies conflicts within the One Health approach has received increasing attention lately, especially in light of emerging global health threats (to humans and animals) and the ongoing environmental and climate changes, including biodiversity challenges. In addition, one aspect of One Health, known as One Welfare (57), has brought ethical aspects closer to the core of the concept. This approach emphasizes not only the physical health of humans and animals but has also added a clearer focus on mental wellbeing, which—at least for humans - certainly involves ethical considerations of how our society influences the welfare of animals and, when expanded, also ethical considerations related to less sentient beings and entire ecosystems. This must be considered when further developing the ethical approaches to be integrated into the One Health concept.

Future studies should explore how these ethical decision models impact the practice of One Health and the SDGs. While ethical issues related to One Health approaches and the fulfillment of SDGs have been discussed (58), little attention has been paid to ethical frameworks for approaching these issues.

## 8 Conclusion

This article illustrates how ethics can be integrated into One Health approaches, emphasizing the need for the field to move beyond merely advocating for ethics to firmly establish it as a core aspect of research. To be effective, ethical decision-making models must be analyzed, developed, and established within One Health approaches to facilitate solutions to wicked problems that frequently arise in this domain.

## Data Availability

The original contributions presented in the study are included in the article, further inquiries can be directed to the corresponding author.

## Footnotes

^1^International Sustainable Development Research Society.

^2^Held in Stockholm, June 2022, https://2022.isdrsconferences.org/10d-one-health-sdgs-and-ethical-conflicts/

^3^ https://dictionary.apa.org/resilience
